# Supplementary material for: Content-rich biological network constructed by mining PubMed abstracts
Source: BMC Bioinformatics. 2004 Oct 8;5:147. doi: 10.1186/1471-2105-5-147 (PMC528731; doi:10.1186/1471-2105-5-147)
Supplement: Additional File 2 — The original results of the above study (non-essential files are deleted to keep the file size under the limit set by BMC bioinformatics). [file 1471-2105-5-147-S2.bz2 › chilibotAdditionalFile2/dip20/143ID7739537E503/html/TFDP2_RB1.html]

 


 **TFDP2** and **RB1** 
  
Found 19 abstracts in PubMed, retrieved 19.  
 

 What does Google say? 
 PDF only 
| .edu only 

---

**Interactive relationship** (e.g. stimulation, inhibition, etc)

**Stimulatory relationship**- Low density DNA array analysis revealed that telomerase activity increases the expression of G1 regulating genes including cyclin D3, cyclin E1, E2F 4, and DP2  [ **TFDP2** ] , associated with hyperphosphorylation of retinoblastoma  [ **RB1** ] , leading to the extended proliferative capacity of BMSSC Ts.  Ref: 12674332 J Bone Miner Res, 2003
**Neutral relationship**

**Non-interactive relationship** (e.g. studied together, co-existance, homology, etc.)

- To elucidate the biochemical processes involved in the pathogenesis of B CLL and MCL, we analyzed the expression level of a set of genes that play central roles in apoptotic or cell proliferation pathways and of candidate genes from frequently altered genomic regions, namely ATM, BAX, BCL2, CCND1, CCND3, CDK2, CDK4, CDKN1A, CDKN1B, E2F1, ETV5, MYC,  **RB1** , SELL,  **TFDP2** , TNFSF10, and TP53.  Ref: 12036888 Blood, 2002
